# Validation of the Kinematic Assessment Protocol Used in the Technology-Supported Neurorehabilitation System, Rehabilitation Technologies for Hand and Arm (R3THA™), in Children and Teenagers with Cerebral Palsy

**DOI:** 10.3390/s24155013

**Published:** 2024-08-02

**Authors:** Qinyin Qiu, Ashley J. Mont, Amanda Gross, Gerard Fluet, Sergei Adamovich, Mee Eriksson

**Affiliations:** 1 NeuroTechR3, Inc., 211 Warren Street, Newark, NJ 07103, USA; am864@njit.edu (A.J.M.);; 2 Rutgers Biomedical and Health Sciences, Rutgers University, 65 Bergen St, Newark, NJ 07107, USA; fluetge@shp.rutgers.edu; 3 Department of Biomedical Engineering, New Jersey Institute of Technology, 323 Dr Martin Luther King Jr Blvd, Newark, NJ 07102, USA; sergei.adamovich@njit.edu

**Keywords:** children and teenagers with cerebral palsy, motor rehabilitation, exergame, upper extremity assessment

## Abstract

This study evaluates the R3THA™ assessment protocol (R3THA-AP™), a technology-supported testing module for personalized rehabilitation in children with cerebral palsy (CP). It focuses on the reliability and validity of the R3THA-AP in assessing hand and arm function, by comparing kinematic assessments with standard clinical assessments. Conducted during a 4-week summer camp, the study assessed the functional and impairment levels of children with CP aged 3–18. The findings suggest that R3THA is more reliable for children aged 8 and older, indicating that age significantly influences the protocol’s effectiveness. The results also showed that the R3THA-AP’s kinematic measurements of hand and wrist movements are positively correlated with the Box and Blocks Test Index (BBTI), reflecting hand function and dexterity. Additionally, the R3THA-AP’s accuracy metrics for hand and wrist activities align with the Melbourne Assessment 2’s Range of Motion (MA2-ROM) scores, suggesting a meaningful relationship between R3THA-AP data and clinical assessments of motor skills. However, no significant correlations were observed between the R3THA-AP and MA2’s accuracy and dexterity measurements, indicating areas for further research. These findings validate the R3THA-AP’s utility in assessing motor abilities in CP patients, supporting its integration into clinical practice.

## 1. Introduction

### 1.1. Cerebral Palsy (CP) Background

Cerebral palsy (CP) is a non-progressive neurological disorder that affects muscle control, movement, cognition, perception, and coordination [1]. Improving manual skills is an important treatment goal for children and teenagers with CP because of the upper extremities’ central role in daily living skills [2]. The development of manual skills necessitates a large volume of focused training [3]. Unfortunately, the quality and volume of rehabilitation does not often meet the requirements of children and teenagers with CP, due to the space and personnel costs associated with inpatient care and the transportation and access issues associated with outpatient rehabilitation.

### 1.2. Telerehabilitation in CP

Telerehabilitation is an approach to rehabilitation designed to improve patient access and limit costs to providers. Telerehabilitation consists of remote assessment, remote treatment, and telemonitoring. These tasks are accomplished by capturing patient movement using a variety of interfaces which include cameras, electromyography, data gloves, and force sensors, and then transmitting the movement data to clinicians via telecommunications equipment [4,5,6].

**Figure 1:**
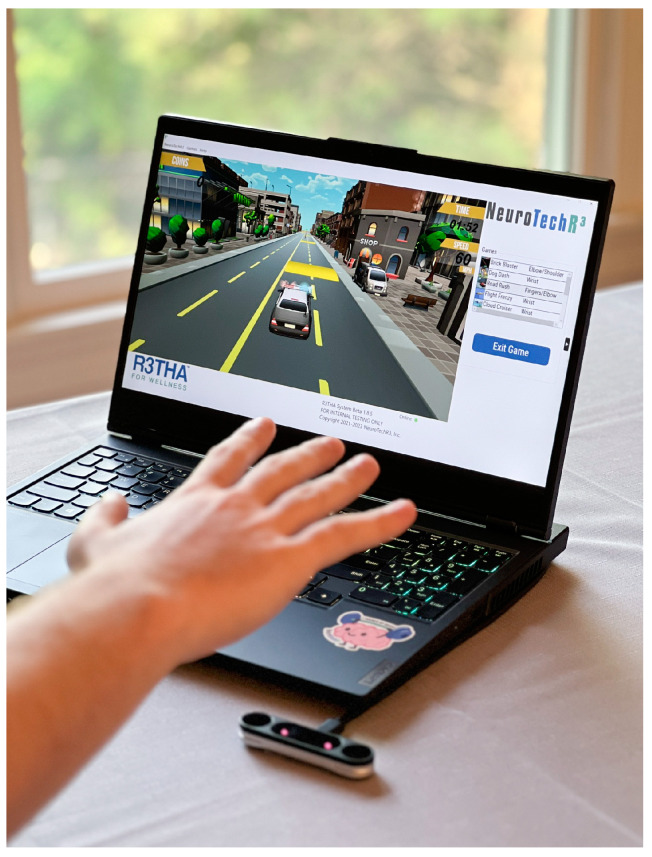
Photograph of a subject playing one of the exergames in R3THA.

One example of a telerehabilitation system is the Rehabilitation Technologies for Hand and Arm (R3THA™) system, which uses exergaming technologies to provide hand and arm rehabilitation activities for individuals with central nervous system dysfunction (Figure 1). R3THA is a camera-based telerehabilitation system, specifically designed to work without wearable sensors attached to the hand. It uses an infrared camera, the Ultraleap Leap Motion Controller 2 (ULMC2), to capture kinematic measurements. The ULMC2 has two infrared light-emitting diodes (LEDs) that collect images transmitted via USB to the ULMC2’s tracking software, which analyzes and transforms the images into three-dimensional representations [7]. This allows for real-time, camera-based estimation of wrist and finger angles and position measurements. R3THA also provides a comprehensive assessment protocol (R3THA-AP), which collects a battery of kinematic measurements. A previous iteration of this approach, the Home-Based Virtual Rehabilitation System (HoVRS), developed by the New Jersey Institute of Technology (NJIT), was originally designed for studying individuals recovering from stroke [7].

### 1.3. Challenges in Remote Assessment in Children and Teenagers with CP

Of the three aspects of telerehabilitation (remote assessment, remote treatment, and telemonitoring), remote assessment is particularly challenging in children and teenagers with CP due to difficulties in collecting valid, objective measurements of motor function, particularly in smaller and younger children [8]. With this being said, fine-grained, objective measurements are especially important in younger children, where changes in motor performance might be small and hard to detect for clinicians and caregivers [9]. Standardized computer-based assessments can provide objective and instantaneous evaluation of the upper extremities’ function and make remote and more frequent monitoring possible. However, in order to be feasible, these assessments need to accommodate the anthropometrics and cognitive perceptual/abilities of the children being tested. 

### 1.4. Study Objectives

Studies show that camera-based motion capture systems are reliable and effective for the provision of upper extremity therapy activities for children and teenagers with CP in laboratory settings [10,11,12]. However, a higher degree of precision is required for remote assessments to be sufficiently reliable and valid for measuring progress and facilitating clinical decision making by therapists. Systems that utilize camera-based gesture recognition require usable anthropometric data for transformation into a hand model that can be used for kinematic measurement. Hand shape and size affect these measurements. To date, no acceptable range of hand sizes for utilization in camera-based motion capture has been established. 

Study One in this submission will attempt to determine whether there is a minimum upper extremity size, as measured by elbow crease to fingertip measurements, necessary for the R3THA system to collect valid kinematic measurements. 

Remote motor assessment also requires a degree of direction-following and attention beyond those of standardized assessments presented in person [13]. Complex sensorimotor transformations are also necessary for users to understand the translation of physical movement to on-screen movement [14]. All these abilities are impacted by the neuropathology associated with CP and the developmental process. There are no established guidelines describing an ideal age range or minimum age for the collection of valid, camera-based motor performance data in children and teenagers with CP. Study One in this submission will also attempt to determine whether there is a minimum age necessary for therapists using the R3THA system to collect valid kinematic measurements.

As stated previously, the ULMC2 needs to be able to detect several key anthropometric landmarks in order to recognize an upper extremity and convert the movement of the upper extremity into kinematic data. Certain hand positions obscure these landmarks, resulting in invalid kinematic data. Previous research has identified specific movement impairments that are associated with difficulties in obtaining valid kinematic data using the ULMC2 [15,16]. R3THA-AP-based measurements, conducted both in person and remotely, have demonstrated clinically acceptable levels of reliability as well as moderate to strong correlations with clinical measurements of motor control in stroke patients. This suggests that the R3THA-AP can generate the meaningful remote assessments necessary for effective telerehabilitation [17].

Study Two in this submission will present a preliminary pilot study examining the correlations between tests of upper extremity function performed on a group of children and teenagers with CP and the six kinematic measurements contained in the R3THA-AP, to determine which of the tests is able to collect kinematic data that is associated with real world function. Taken together, these two pilot studies will be used to inform a definitive study focusing on the clinometrics of remote assessments produced for children and teenagers with CP using the R3THA-AP.

## 2. Study One: Exploring the Relationship between R3HTA-AP Measurement Data and Children’s Ages and Upper Extremity Sizes

### 2.1. Materials and Methods

#### 2.1.1. Study Participants

Both studies were approved by the Internal Review Board of the New Jersey Institute of Technology. Children were recruited at a summer camp in suburban New Jersey, USA. Children from across the US attend this summer camp, traveling to New Jersey for the four-week duration of the camp. Parents and children were informed about the study on the first day of camp. Potential participants were pre-screened by a camp Occupational Therapist (OT) and an on-site study coordinator. Consent and assent were obtained from parents and children.

Participants were selected for both studies based on the following inclusion criteria: (1) children with CP or hemiplegia due to brain injury; (2) aged 3–18; (3) Gross Motor Function Classification System level of I–IV; (4) no severe arm weakness or tone; (5) able to follow verbal instructions; (6) no visual problems that make it difficult for them to interact with the entire computer screen.

#### 2.1.2. Study Protocol

To prepare for the study, study therapists were trained to use the R3THA-AP a week before the summer camp started. Therapists received an initial 2-h training session focused on understanding the core functionalities of R3THA, including how to operate the software, set up and standardize the hardware positioning, and troubleshoot common issues. Following the initial training, therapists engaged in practical sessions where they interacted with the system as users to gain first-hand experience. Finally, the therapists conducted a 60-min session with two children with CP using R3THA under the supervision of a study technologist. After consent and assent was obtained, each participant’s motor function was evaluated using clinical assessments and R3THA-AP-based kinematic assessments during the first week of the camp by four licensed OTs.

#### 2.1.3. Data Collection

##### Demographic Data

Demographic information, such as the participants’ age, gender, and side of hemiplegia, was gathered before the study commenced. Each participant’s upper extremity size was measured from the tip of the middle finger to the elbow crease.

##### R3THA Assessment

The R3THA system utilizes the ULMC 2, which internally combines three infrared LEDs and two cameras. The cameras collect data from a conical field (apex down) beginning 10 cm above the device and ending 60 cm above the device. The ULMC 2’s cameras collect hand images at a frequency of 120 Hz. The LEDs pulsate near-infrared light at the same frequency, amplifying the camera’s ability to capture objects that are directly illuminated by the LEDs. The ULMC 2 streams the resulting greyscale image of the near-infrared light spectrum to its USB controller, which reads the position of anatomical landmarks directly into its local memory, adjusting the resolution as needed. After this, data streams to the Leap Motion Image Application Programming Interface (API) via USB. From the API, R3THA feeds anatomical landmark position data to Unity to produce avatar movement that interacts with on-screen testing activities by calling the Leap Motion API.

The R3THA-AP (R3THA Assessment Protocol) collects a battery of kinematic measurements to assess the range of motion and the control of movements in the hand and arm (please see MontJohnson et al. [17] for an in-depth description of R3THA-AP procedures). Table 1 describes the R3THA-AP measurements.

#### 2.1.4. Statistical Analysis

We explored the relationship between R3THA-AP’s measurements and the participants’ ages and upper extremity sizes using Spearman rank-order correlations (SROC). Spearman’s coefficients between 0.02 and 0.39 were considered weak and coefficients between 0.40 and 0.69 were considered moderate [18]. SROC were also utilized to determine the correlation between upper extremity size and percentage of valid R3THA-AP data. The statistical data analyses were conducted using R 4.1.0 and a custom-written script in MATLAB software (MathWorks, Natick, MA, USA, v.R2022b).

### 2.2. Results

#### 2.2.1. Participants

A total of 36 participants between 3 and 18 years old were recruited to evaluate the usability of the R3THA-AP for children and teenagers with CP. This broad range of ages and hand sizes was chosen in order to identify the age and hand size cutoffs associated with valid measurements.

Consent and assent were obtained from each participant prior to being enrolled in the study. The demographic characteristics are listed in Table 2. No safety issues were reported, and none of the participants experienced any side effects during the study. A child with CP is using R3THA-AP to assess the accuracy of wrist extension and flexion is shown in the Supplementary Video S1.

#### 2.2.2. Data Validation Analysis

**Figure 2:**
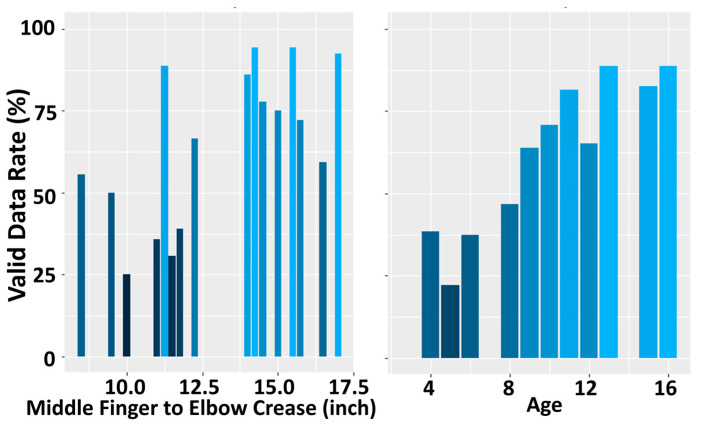
Distribution of valid measurements collected by upper extremity length (**left** panel) and age (**right** panel). Note the difference in the percentage of valid measurements for subjects greater than eight years of age.

During the study, if the system was unable to track the participant’s hand for a specific subtest, the corresponding R3THA-AP subtest item score was recorded as null. The R3THA-AP valid data rate was calculated as the percentage of non-null R3THA-AP kinematic measurements relative to the total number of subtests. R3THA-AP’s rate of valid data collected from participants aged 8 and older is 40% higher than that collected from participants aged 7 and younger. The valid data rate is statistically significantly correlated with age (Spearman’s ρ = 0.94, *p*-value < 0.001), and moderately correlated with upper extremity size (Spearman’s ρ = 0.602, *p*-value = 0.013) (Figure 2).

## 3. Study Two: Evaluating the Validity of the R3HTA-AP by Correlating Its Kinematic Measurements with Clinical Assessments

### 3.1. Materials and Methods

#### 3.1.1. Study Participants

See Section 2.1.1.

#### 3.1.2. Study Protocol

To prepare for the study, study therapists were trained to use the R3THA-AP a week before the summer camp started (see Section 2.1.2). After consent and assent were obtained, the participants’ motor function was evaluated using clinical assessments and R3THA-AP-based kinematic assessments during camp by four licensed OTs.

#### 3.1.3. Data Collection

##### Demographic Data

Demographic information, such as participants’ age, gender, and side of hemiplegia, was gathered before training commenced.

##### Clinical Assessments

The Melbourne Assessment 2 (MA2): MA2 is a 14-item criterion-referenced test for evaluating four elements of upper limb movement quality in children with a neurological impairment aged 2.5 to 15 years: (i) range of movement; (ii) accuracy of reach and placement; (iii) dexterity of grasp, release, and manipulation; and (iv) fluency of movement [19]. MA2 performance is video recorded and scored after the performance is complete.

Box and Block Test (BBT): BBT is a standardized activity level test used to measure gross manual dexterity [20,21]. The subject is given 60 s to move as many blocks as possible over a partition to the other side, using one hand. Subjects perform the test once with each hand. The Box and Blocks Index (BBTI) is calculated by subtracting the more impaired hand’s score from the less impaired hand’s score.

#### 3.1.4. R3THA Assessment

See Section 2.1.3.

### 3.2. Statistical Analysis

The values for kinematic measurements, along with MA2 and BBTI, were analyzed to explore the relationship between the kinematic measurements provided by LMC and the level of impairment assessed clinically. SROC were utilized to determine the correlation between clinical assessments and each kinematic measurement from the R3THA-AP.

### 3.3. Results

#### 3.3.1. Participants

The R3THA-AP data set of 21 participants from Study One was used for this study. These 21 participants were selected based on the following criteria: (1) aged 8 years or older, (2) valid data rate is at 65% or above. The demographic characteristics and initial clinical assessment results are listed in Table 3. No safety issues were reported, and none of the participants experienced any side effects during the study.

#### 3.3.2. Correlation Analysis

**Figure 3:**
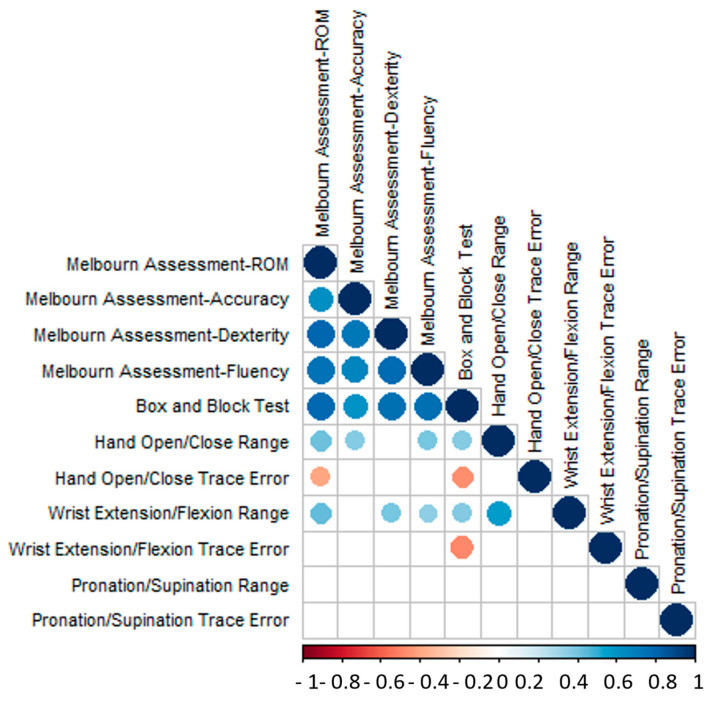
Correlation matrix between Box and Block Test, Melbourne Assessment-ROM, Melbourne Assessment-Accuracy, Melbourne Assessment-Dexterity, Melbourne Assessment-Fluency, and each kinematics measurement from R3THA-AP. Pairs that do not have a significant coefficient (*p*-value ≤ 0.05) are left blank.

The correlation matrix between the pairs of kinematic measurements from the R3THA-AP and BBTI and all four categories of MA2 is shown in Figure 3. Only the significantly correlated pairs (*p* ≤ 0.05) are illustrated with circles.

##### Correlation between Box and Blocks Test and R3THA-AP

**Figure 4:**
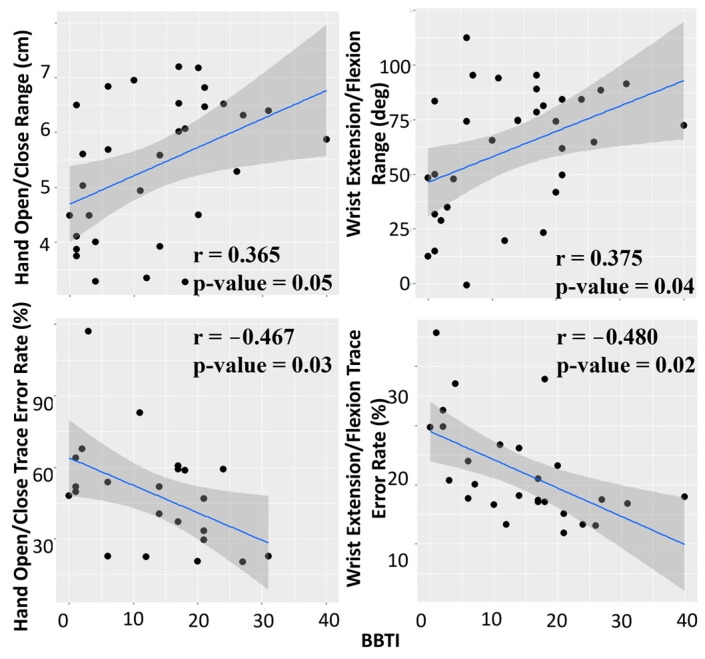
Hand Open/Close Range and Wrist Extension/Flexion Range show a statistically significant correlation with BBTI, as does the trace error rate using Hand Open/Close and Wrist Extension/Flexion.

The Hand Open/Close Range and Wrist Pitch Range show a positive correlation with BBTI (Figure 4). The Hand Open/Close Tracing Error and Wrist Pitch Tracing Error show a negative correlation with BBTI (higher BBTI score, less trace error = higher accuracy). The two measurements of pronation/supination did not demonstrate statistically significant correlations with BBTI scores.

##### Correlation between MA2-ROM and R3THA-AP

**Figure 5:**
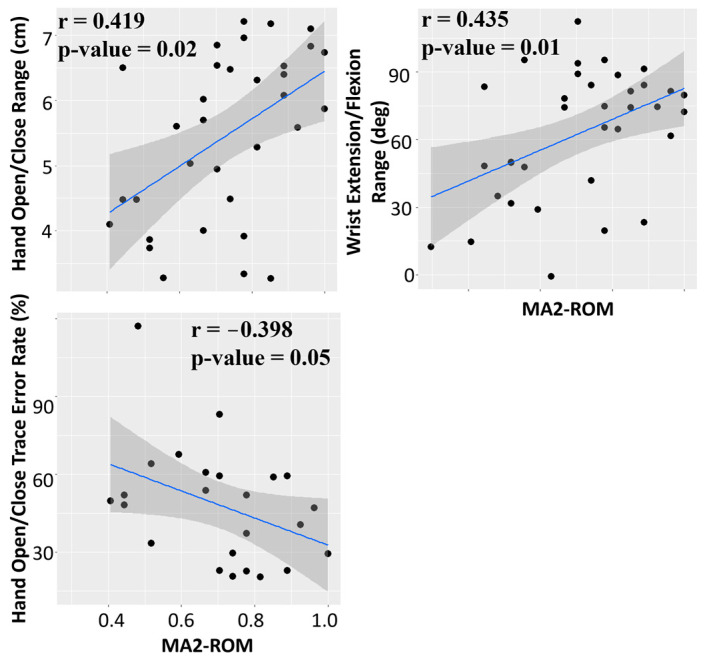
Melbourn Assessment 2’s Range of Motion shows a statistically significant correlation with the R3THA-AP Hand Open/Close Range, Wrist Extension/Flexion Range, and Hand Open/Close Tracing Error Rate.

Hand Open Range, Wrist Pitch Range, and Wrist Extension/Flexion Accuracy demonstrated statistically significant correlations with MA2-ROM (Figure 5).

##### Correlation of R3THA-AP with MA2-Accuracy and MA2-Dexterity

**Figure 6:**
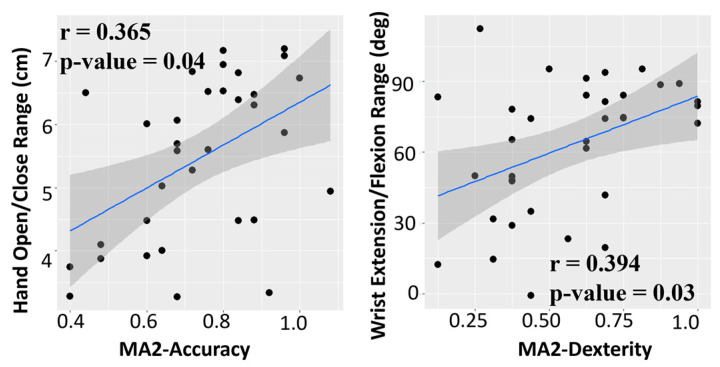
MA2-Accuracy is correlated with Hand Open/Close Range. MA2-Dexterity is correlated with Wrist Extension/Flexion Range.

R3THA’s Hand Open Range measurements demonstrated statistically significant correlations with MA2-Accuracy. R3THA’s Wrist Extension/Flexion Range measurements demonstrated statistically significant correlations with MA2-Dexterity (Figure 6).

## 4. Discussion

Our study found that age was a stronger predictor of the ability to collect usable kinematic data with the R3THA than upper extremity size in a group of children with CP. While age and upper extremity size were highly correlated with each other, the ability to collect valid kinematic data did not increase linearly with upper extremity size or demonstrate a clear benchmark, which is consistent with the findings of a study of the measurement characteristics of the Leap Motion Controller by Chan [22].

However, subjects under eight years of age in this study had substantial difficulty performing the assessments contained in the R3THA-AP, and subjects eight years and older were able to successfully perform these tests at a much higher frequency. This may suggest that developmental issues impact the validity of data produced by the R3THA-AP and that age 8 might be a suitable benchmark for achieving usable measurements.

These findings are congruent with the findings of a systematic review by Martinie [23] and consistent with the neurodevelopmental literature, which states that children typically develop the functional visual and cognitive skills necessary to manipulate, organize, and prioritize objects and actions, along with the ability to concentrate, by the age of 8 [24].

The R3THA-AP was able to collect valid measurements of finger and wrist movement in a majority of the children tested in Study One, consistent with the published literature [25]. However, the number of valid data sets for the measurement of Pronation/Supination Range and Trace was low, and we did not find correlations between R3THA-based measurements of pronation/supination and clinical measurements of motor function in Study Two. This could be due to the characteristic of upper extremity impairment in children with CP. An excessive inward rotation of the forearm, known as hyper forearm pronation, is common in children with CP, particularly in those with spastic types of CP [26]. The motion capture camera used by R3THA faces challenges in tracking and assessing the hand in this position because it relies on the visibility of all five fingers to recognize a hand. When the hand is in a hyper-pronated position, it becomes difficult for the camera to detect it accurately if the camera is placed on a table. These findings are consistent with those described by Smeragliuolo, who cited problems with LMC-based measurement of pronation/supination [25]. Future implementation of the ability to rotate the camera to directly face the palm or utilize an array of multiple cameras to improve the ability to track pronation/supination movements is part of the development and future study plan for R3THA.

In Study Two, four of the six measurements provided by the R3THA-AP demonstrated statistically significant correlations with activity-based clinical assessments. Sub-items of the R3THA-AP measurements, such as Hand Open/Close Range, Hand Open/Close Trace Error, Wrist Flexion/Extension Range, and Wrist Flexion/Extension Trace Error, showed weak to moderate statistically significant correlations with BBT, and weak to moderate statistically significant correlations with the ROM, Accuracy, and Dexterity measurements produced by the MA2. There are no published studies on correlations between camera-based measurements of wrist and finger kinematics and those obtained with BBT or elements of the MA2, but these correlations are comparable to, or slightly lower than, correlations between computerized tests of hand function and activity level clinical tests in adults [27,28]. This suggests that R3THA-based measurements may provide valid assessments of motor skills and abilities in children with CP.

The wide age range and relatively small sample size of this exploratory study limit the generalizability of our findings to persons of a specific age. Further study with a larger sample size will be necessary to draw definitive conclusions. This study was conducted at a summer camp for children with CP. This would imply that the children in our sample have had more access to rehabilitation than many children with CP, which decreases the generalizability of our findings to children from underserved populations.

## 5. Conclusions

This study found that age was a stronger predictor of the ability to collect usable kinematic data with the R3THA-AP than upper extremity size in a group of children with CP. Measurements of hand opening and wrist range of motion, as well as measurements of wrist and finger tracking movements, provided by the R3THA-AP demonstrated statistically significant correlations with standardized, activity-based clinical assessments. This in-person proof of concept study suggests that the system can generate the valid assessments necessary for effective telerehabilitation. Future study with remote subjects is indicated.

## 6. Patent

Eriksson M., Qiu Q., Cronce A., MontJohnson A., inventors; NeuroTechR3 Inc., assignee. SYSTEM AND METHOD FOR PERFORMING REHABILITATION EXERCISES. US patent application 63538935, 18 September 2023.

## Figures and Tables

**Table 1:** R3THA-AP kinematic measurements and descriptions.

R3THA-AP Subtest Items	Descriptions	Equation
Hand Open/Close Range (cm)	The participant fully opens and then fully closes their hand. The Hand Open/Close Range value is calculated by measuring the difference in the average distance between the fingertips and the center of the palm across all four fingers in these two positions. A larger value indicates a greater hand opening range.	$\frac{\sum_{n=1}^{5}{D\_handopen}_{n}-\sum_{n=1}^{5}{D\_handclose}_{n}}{5}$ where ${D\_handopen}_{n}$ is the distance between the nth fingertips and the center of the palm when the hand is open; ${D\_handclose}_{n}$ is the distance between the nth fingertips and the center of the palm when the hand is closed.
Hand Open/Close Trace Error Rate (%)	The participant controls a cursor that moves up and down by opening and closing their hand. The participant attempts to trace an irregular wave which moves on the screen from left to right at a constant speed. The trace error rate is calculated as the root mean square error (RMSE) between the cursor position and the corresponding target point on the wave normalized by Hand Open/Close Range. The smaller the value, the better the control of hand opening.	$\sqrt{\frac{1}{n}\sum_{i=1}^{n}{\frac{\left(y_{i}-{\overbrace{y}}_{i}\right)}{Hand\;Range}}^{2}}$ where *n* is the number of observations; $y_{i}$ is the cursor position; ${\overbrace{y}}_{i}$ is the corresponding target point on the wave; Hand Range is the Hand Open/Close Range.
Wrist Extension/Flexion Range (deg)	The participant extends and flexes their wrist against gravity with their forearm in a fixed position. The angular difference between these two positions is reported as the wrist pitch range.	Wrist Extension/Flexion Range = Max Wrist Extension angle + Max Wrist Flexion angle
Wrist Extension/Flexion Trace Error Rate (%)	The participant controls a cursor that moves up and down by extending and flexing their wrist. They use the cursor to trace a sine wave on the screen. The trace error rate is calculated as the root mean square error (RMSE) between the cursor position and the corresponding target point on the wave.	$\sqrt{\frac{1}{n}\sum_{i=1}^{n}{\frac{\left(y_{i}-{\overbrace{y}}_{i}\right)}{Wrist\;Range}}^{2}}$ where *n* is the number of observations; $y_{i}$ is the cursor position; ${\overbrace{y}}_{i}$ is the corresponding target point on the wave; Wrist Range is the wrist extension/flexion range.
Pronation/Supination Range (deg)	The participant moves and holds their hand in pronation and supination with their elbow fixed. The range is then calculated.	Pronation/Supination Range = Max Pronation angle + Max Supination angle
Pronation/Supination Trace Error Rate (%)	The participant controls a cursor that moves up and down by pronating and supinating their hand. They use the cursor to trace a sine wave on the screen. The trace error rate is calculated as the root mean square error (RMSE) between the cursor position and the corresponding target point on the wave.	$\sqrt{\frac{1}{n}\sum_{i=1}^{n}{\frac{\left(y_{i}-{\overbrace{y}}_{i}\right)}{Roll\;Range}}^{2}}$ where *n* is the number of observations; $y_{i}$ is the cursor position; ${\overbrace{y}}_{i}$ is the corresponding target point on the wave; Roll Range is the forearm pronation/supination range.

**Table 2:** Demographics (N = 36).

	Mean (SD)
Age	9.28 (4.4)
Gender	15 females/21 males
Hemiplegia side	17 left/19 right
Upper extremity size (inches)	13.22 (2.53)

**Table 3:** Demographics (N = 21).

	Mean (SD)
Age	11.89 (2.5)
Gender	7 females/14 males
Hemiplegia side	11 left/9 right
Initial Box and Block Test	13.15 (10.13)
Initial MA2-ROM	0.71 (0.18)
Initial MA2-Accuracy	0.57 (0.24)
Initial MA2-Dexterity	0.74 (0.24)
Initial MA2-Fluency	0.62 (0.16)

## Data Availability

De-identified data will be supplied by the corresponding author if requested in writing.

## Supplementary Materials

The following supporting information can be downloaded at: https://www.mdpi.com/article/10.3390/s24155013/s1.
